# Supplementary material for: Impact of Early Mobilization on Recovery after Major Head and Neck Surgery with Free Flap Reconstruction
Source: Cancers (Basel). 2021 Jun 8;13(12):2852. doi: 10.3390/cancers13122852 (PMC8227616; doi:10.3390/cancers13122852)
Supplement: Supplementary file 1 [file cancers-13-02852-s001.zip › cancers-1213905-supplementary.pdf]

Table S1. Patient characteristics and missing data

Characteristic	Pathway Data (%) *n*=445	Missing Data (%) *n*=55
**Sex**		
Male	303 (68)	39 (71)
Female	142 (32)	16 (29)
**Age (years)**		
Mean ± SD	61.2 ± 12.2	60.2 ± 12.7
Range	21.2 – 89.0	22.2 – 83.4
**Alcohol status**		
Never	90 (20)	6 (11)
Light/Moderate	162 (36)	15 (27)
Heavy	93 (21)	21 (38)
Former	48 (11)	6 (11)
Not reported	52 (12)	7 (13)
**Smoking status**		
Never smoked	117 (26)	13 (24)
Former smoker	151 (34)	23 (42)
Current smoker	136 (31)	15 (27)
Not reported	41 (9)	4 (7)
**Primary site**		
Oral cavity	303 (68)	30 (55)
Pharynx & Larynx	42 (8)	8 (15)
Skin	39 (9)	7 (13)
Paranasal/Nasal	27 (6)	4 (7)
Other	34 (8)	6 (11)
**Histology**		
Squamous cell	356 (80)	41 (75)
Other cancer	83 (18)	10 (18)
Benign	6 (1)	3 (5)
Not reported	7 (2)	1 (2)
**Clinical stage**		
0	8 (2)	1 (2)
I	44 (10)	0 (0)
II	70 (16)	3 (5)
III	66 (15)	6 (11)
IV	223 (50)	35 (64)
Not reported	34 (8)	10 (18)
**Number of free flaps**		
One	423 (95)	52 (95)
Two	22 (5)	3 (5)
**Flap type**		
Radial forearm	235 (53)	21 (38)
Fibula	95 (21)	9 (16)
Anterolateral thigh	57 (13)	17 (31)
Other	58 (13)	8 (15)
**Resection extent**		
Soft tissue	329 (74)	43 (78)
Bone	97 (22)	9 (16)
Soft tissue & bone	19 (4)	3 (6)

Values may not sum to 100% due to rounding

Table S2. Patient characteristics and mobilization within or after 48 h

Characteristic	POD 0-2 (%) *n*=342	After POD 2 (%) *n*=103	*p*-value
**Sex**			
Male	235 (68)	68 (66)	0.630
Female	107 (32)	35 (34)	
**Age (years)**			
Mean ± SD	61.2 ± 12.2	61.1 ± 12.2	0.482*
**Alcohol status**			
Never	66 (22)	24 (27)	0.391
Light/Moderate	134 (44)	28 (31)	
Heavy	68 (22)	25 (28)	
Former	35 (12)	13 (14)	
**Smoking status**			
Never smoked	95 (31)	22 (24)	0.267
Former smoker	110 (35)	41 (44)	
Current smoker	106 (34)	30 (32)	
**Comorbidities**			
None	106 (31)	36 (35)	0.185
One	112 (33)	24 (23)	
Two or more	124 (36)	43 (42)	
**Specific Comorbidity**			
Diabetes	39 (11)	15 (15)	0.392
COPD	39 (11)	11 (11)	1.000
Hypertension	136 (39)	45 (44)	0.494
Heart disease	37 (11)	22 (21)	**0.00**8
**Primary site**			
Oral cavity	230 (67)	73 (71)	0.279
Pharynx & Larynx	33 (10)	9 (9)	
Skin	35 (10)	4 (4)	
Paranasal/Nasal	19 (6)	8 (8)	
Other	25 (7)	9 (9)	
**Histology**			
Squamous cell	272 (81)	84 (82)	**0.040**
Other cancer	58 (17)	18 (18)	
Benign	6 (2)	0 (0)	
**Clinical stage**			
0	4 (1)	4 (4)	0.151
I	35 (11)	9 (9)	
II	57 (18)	13 (13)	
III	53 (17)	13 (13)	
IV	163 (52)	60 (61)	
**Number of free flaps**			
One	328 (96)	95 (92)	0.191
Two	14 (4)	8 (8)	
**Flap type**			
Radial forearm	183 (53)	52 (50)	0.749
Fibula	69 (20)	26 (25)	
Anterolateral thigh	45 (13)	12 (12)	
Other	45 (13)	13 (13)	
**Resection extent**			
Soft tissue	261 (76)	68 (66)	**0.047**
Bone	70 (21)	27 (26)	

Soft tissue & bone	11 (3)	8 (8)

Values may not sum to 100% due to rounding. Fisher's exact tests were used for p-values, except for age (*independent t-test).

Table S3. Predictors of any postoperative complication and major postoperative complications using elastic net regularization followed by multivariable logistic regression.

**Characteristic** *(reference level)*	**Any complication**				**Major complications**			
	**OR**	**95% CI**	*z*	***p*-value**	**OR**	**95% CI**	*z*	***p*-value**
**Age**	1.02	1.00-1.05	2.18	**0.029**	1.01	0.98-1.04	0.88	0.377
**BMI category** *(healthy range)*								
Underweight	2.70	0.91-8.06	1.78	0.075	1.88	0.54-6.49	1.00	0.318
Overweight	0.69	0.39-1.23	-1.27	0.206	0.50	0.22-1.12	-1.68	0.093
Obese	1.04	0.54-2.02	0.13	0.899	0.82	0.36-1.92	-0.45	0.654
**Sex** *(male)*								
Female	0.75	0.43-1.30	-1.02	0.309	0.66	0.31-1.42	-1.05	0.292
**Smoking Status** *(never)*								
Former smoker	0.63	0.32-1.21	-1.39	0.166	0.47	0.20-1.10	-1.74	0.081
Current smoker	0.99	0.47-2.07	-0.04	0.969	0.21	0.07-0.59	-2.96	**0.003**
**Alcohol status** *(never)*								
Light/Moderate	0.72	0.37-1.42	-0.94	0.347	1.89	0.76-4.73	-1.37	0.171
Heavy	0.67	0.29-1.54	-0.94	0.349	1.23	0.38-3.96	0.35	0.725
Former drinker	0.41	0.16-1.03	-1.89	0.059	0.48	0.11-2.08	-0.99	0.324
**Comorbidity** *(none)*								
One	2.13	1.17-3.88	2.46	**0.014**	1.68	0.68-4.19	1.12	0.263
Two or more	1.93	1.06-3.54	2.13	**0.033**	2.57	1.10-6.00	2.18	**0.029**
**Primary Site** *(oral cavity)*								
Pharynx& Larynx	1.55	0.67-3.63	1.01	0.311	1.50	0.52-4.29	0.76	0.449
Paranasal/Nasal	0.67	0.20-2.18	-0.67	0.502	0.30	0.05-1.74	-1.34	0.180
Skin	1.15	0.35-3.72	0.23	0.819	0.20	0.03-1.58	-1.53	0.127
Other	0.37	0.11-1.28	-1.57	0.117	0.32	0.05-1.92	-1.25	0.211
**Cancer stage** *(I-II)*								
III-IV	1.96	1.15-3.35	2.47	**0.013**	2.22	0.96-5.14	1.86	0.063
**Flap count** *(one)*								
Two	1.09	0.06-21.05	0.06	0.956				
**Resection extent** *(soft tissue only)*								
Bone			0.02	0.986				
Soft tissue & bone	2.50	0.10-62.69	0.56	0.577				
**Flap donor type** *(radial forearm)*								
Fibula			-0.02	0.986	1.19	0.48-2.92	0.37	0.710
Anterolateral thigh	0.96	0.44-2.07	-0.11	0.909	1.41	0.47-4.29	0.61	0.541
Other	1.61	0.66-3.93	1.05	0.292	3.42	1.40-8.34	2.70	**0.007**
**Unit arrival** *(POD 0-1)*								
After POD 1	1.53	0.73-3.23	1.12	0.262	1.98	0.85-4.61	1.59	0.111
**Mobilization** *(POD 0-1)*								
After POD 1	1.23	0.71-2.12	0.75	0.455	0.97	0.43-2.21	-0.07	0.945
**Mobilization** *(POD 0-2)*								
After POD 2	1.75	0.88-3.50	1.59	0.111	2.61	1.10-6.21	2.17	**0.030**
**Tracheostomy** *(no)*								
Yes	1.43	0.66-3.11	0.91	0.364	0.38	0.14-1.09	-1.80	0.073

Table S4. Predictors of hospital length of stay using elastic net regularization followed by multivariable logistic regression.

**Characteristic** *(reference level)*	**LOS >10 days**			
	**Odds ratio**	**95% CI**	*z*	***p*-value**
**Age** (centred at the mean)	1.03	1.00-1.05	2.30	0.021
**BMI category** *(healthy range)*				
Underweight	1.11	0.37-3.32	0.18	0.855
Overweight	0.62	0.33-1.19	-1.44	0.151
Obese	0.46	0.22-0.95	-2.10	0.036
**Alcohol status** *(never)*				
Light/Moderate	0.62	0.31-1.27	-1.31	0.191
Heavy	0.76	0.33-1.71	-0.67	0.504
Former drinker	2.43	0.91-6.53	1.76	0.078
**Comorbidity** *(none)*				
One	1.10	0.56-2.19	0.28	0.779
Two or more	1.57	0.77-3.18	1.24	0.215
**Primary Site (***oral cavity)*				
Pharynx & Larynx	5.18	1.80-14.89	3.05	**0.002**
Paranasal/Nasal	0.05	0.01-0.29	-3.34	**0.001**
Skin	0.13	0.03-0.60	-2.60	**0.009**
Other	0.72	0.18-2.86	-0.47	0.640
**Flap count** *(one)*				
Two	0.25	0.01-7.56	-0.80	0.422
**Flap type** *(soft tissue only)*				
Bone	1.08	0.56-2.07	0.22	0.828
Soft tissue & bone	19.93	0.36-1099.16	1.42	0.144
**Mobilization** *(POD 0-2)*				
After POD 2	2.85	1.41-5.76	2.92	**0.004**
**Tracheostomy** *(no)*				
Yes	3.01	1.19-7.65	2.32	**0.020**
**Any complication** *(no)*				
Yes	6.76	3.74-12.22	6.32	**<0.001**
**Major Complication** *(no)*				
Yes	1.97	0.78-4.97	1.44	0.150

Table S5. Predictors of delayed mobilization (after 24 hours) using elastic net regularization followed by multivariable logistic regression.

**Characteristic**	**Delayed mobilization**			
	**Odds ratio**	**95% CI**	***z***	***p*-value**
**Unit arrival** *(POD 0-1)*				
After POD 1	3.29	1.70-6.37	3.52	**<0.001**
**Tracheostomy** *(no)*				
Yes	2.81	1.73-4.56	4.18	**<0.001**
**Any complication** *(no)*				
Yes	1.31	0.86-2.02	1.25	0.213
**Major Complication** *(no)*				
Yes	1.26	0.68-2.35	0.73	0.468
